# Molecular Portrait of an Athlete

**DOI:** 10.3390/diagnostics11061095

**Published:** 2021-06-15

**Authors:** Kristina A. Malsagova, Tatyana V. Butkova, Arthur T. Kopylov, Alexander A. Izotov, Vladimir R. Rudnev, Mikhail S. Klyuchnikov, Alexander A. Stepanov, Anna L. Kaysheva

**Affiliations:** 1 Biobanking Group, Branch of Institute of Biomedical Chemistry “Scientific and Education Center”, 109028 Moscow, Russia; t.butkova@gmail.com (T.V.B.); a.t.kopylov@gmail.com (A.T.K.); farmsale@yandex.ru (A.A.I.); volodyarv@mail.ru (V.R.R.); aleks.a.stepanov@gmail.com (A.A.S.); kaysheva1@gmail.com (A.L.K.); 2 State Research Center Burnasyan of the Federal Medical Biophysical Centre of the Federal Medical Biological Agency of Russia, 123098 Moscow, Russia; kljuchnikov@me.com

**Keywords:** genomics, sports, metabolomics, proteomics, genetic polymorphisms, physical activity, personal training

## Abstract

Sequencing of the human genome and further developments in “omics” technologies have opened up new possibilities in the study of molecular mechanisms underlying athletic performance. It is expected that molecular markers associated with the development and manifestation of physical qualities (speed, strength, endurance, agility, and flexibility) can be successfully used in the selection systems in sports. This includes the choice of sports specialization, optimization of the training process, and assessment of the current functional state of an athlete (such as overtraining). This review summarizes and analyzes the genomic, proteomic, and metabolomic studies conducted in the field of sports medicine.

## 1. Introduction

Sports medicine is the newest field in clinical and theoretical medicine and has significantly impacted the development of the mass physical culture movement (wellness) and elite sports [1]. The field of sports medicine emerged from the accumulative advancements made by physiologists, biochemists, and representatives of other medical and biological disciplines. It is focused on the preventive direction and studies a wide range of independent problems associated with the study of the functional state of an athlete’s body in combination with the clinical criteria of medical control. Physical activity helps to improve the state of the body only if it is adequately correlated with its functional capabilities [2,3]. The main goal of the training process in sports is to achieve the greatest cumulative adaptive effect which should be reflected in an increase in performance indicators, endurance and an improvement in sports results [4]. Under excessive load conditions, a state of fatigue or overtraining develops [5]. Such a state can develop in both beginners and high-class athletes who will require qualified training correction and a monitoring of their recovery process [5,6]. The correctness of the choice of critical values of the duration and intensity of the performed exercise/set of exercises is confirmed by studying the dynamics of the molecular, biomedical, and psychological indicators [7,8,9].

In the modern sports era, there is a continuous increase in the number and level of competitions. This necessitates the improvement of existing methods and the creation of new scientific and methodological recommendations to achieve peak performance during competition while also prolonging the duration of the periods of this peak performance. Taking the scientific achievements, and available sports infrastructure into account, it is necessary to improve the recommendations made to young athletes for the early detection of a predisposition to engage in specific sports by using physiological, medico-biochemical, and molecular genetic methods. Today, the global scientific community is actively working to identify the endogenous molecular factors and processes that can act as personalized predictors of athletic performance [10,11,12].

Proteomic and metabolomic profiling characterizes the state of the body (including features of tissue metabolism and the functioning of body systems) and facilitates the identification of critical changes associated with the body’s response to specific factors. This includes diet, the intake of medications and biological supplements, physical activity, and changes in the psycho-emotional state of an athlete [13,14,15,16]. The dynamics of changes in the qualitative composition and quantitative content of the protein and metabolic factors present in the blood are monitored to aid in the development of an optimal training regimen and to correct the athlete’s diet, supplementation and lifestyle to neutralize the negative factors that hamper the stability needed for sports achievements.

In recent years, a significant part of biomedical research has been aimed at identifying the possible associations between genetic polymorphisms and athletic performance in athletes [17,18,19]. Genetic analysis based on “genome-wide association studies” (GWAS), along with metabolomic and proteomic analyses for sports medicine and hygiene, can be used to outline a prognostic approach if the identified genetic polymorphisms and molecular factors have a strong influence on the metabolic transformation of the athlete’s body [9,20,21]. The relationship between genetic polymorphisms and athletic performance can be confirmed at the protein and metabolic levels. For example, more than 69 genetic markers associated with the status of strength in athletes have been annotated in available scientific literature [20]. Integration of the results of genetic and metabolomic–proteomic analyses with the clinical, biochemical and sports pedagogical indicators will help in the development of personalized recommendations for the effective planning of the athlete training processes.

An analysis of the literature on research related in the field multi-omics for the needs of sports medicine and hygiene was carried out using the PubMed database. The search criteria included the following keywords: “multi-omics” or “genome-wide association studies” or “proteome” or “metabolome” or “genetic polymorphisms” and “sport” or “athletes” or “exercise” or “sports hygiene”. The depth of the literary search was 5 years using additional sources.

The purpose of this review was to analyze the possibility of integrating the results of genetic and metabolomic–proteomic analyses of athletes’ bio-samples with clinical and biochemical parameters.

## 2. Selection of the Type of Biological Material

Most molecular and genetic studies are performed using blood samples which include plasma or serum. This is largely due to the minimally invasive laboratory method of blood collection and the availability of this type of biomaterial. Other biological fluids (cerebrospinal or synovial fluid) and solid samples (tissue biopsies) are of great value in some studies; for example, in identifying the functional characteristics of muscle tissues. However, the procedure for collecting such samples is invasive and requires the participation of a professional doctor. It should be noted that a biomaterial such as urine, which is the least invasive biomaterial available, is frequently used for repeated studies [22]. Advances in molecular biomedicine are constrained by methodological limitations. This is primarily due to the complexity of the composition of different types of biomaterials. Thus, the protein composition of blood is characterized by a dynamic range of more than 10 orders of magnitude. The 20 highly represented blood proteins account for more than 99% (by mass content) of the total blood proteins, which makes it difficult to detect the medium-and low-copy proteins. This limitation can be addressed by using a procedure for the fractionation of biomaterials, depletion of samples from the highly represented proteins, or the enrichment of the target medium- and low-copy proteins. Despite this limitation, plasma currently occupies the leading position among all biological fluids for research in the fields of proteomics and metabolomics.

## 3. Sample Preparation of the Biological Material

The comprehensive assessment of the individual metabolomic, proteomic, and genetic characteristics of an athlete requires the collection of blood, plasma, or serum samples at several points in time during the training process.

Depending on the studied parameters, there are several schemes of the optimal points-of-time for material sampling. These are presented in the diagrams below.

**Figure 1:**
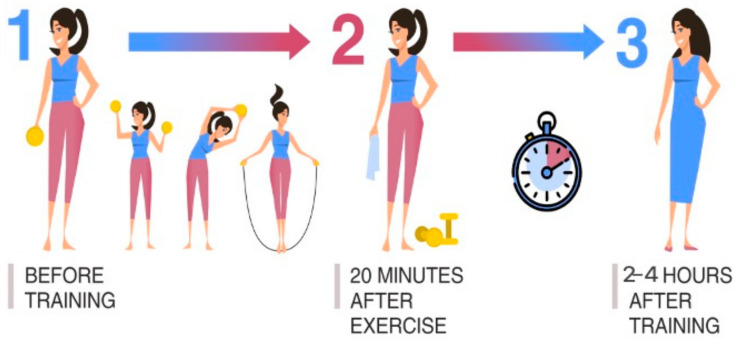
Material sampling is used to assess the recovery ability of the athlete’s body during the training process.

Scheme I is popularly used for assessing the recovery ability of the athlete’s body when leaving the training process. An analysis of the dynamics of body processes, during and after physical activity, is carried out. The nature of the loads is recommended to be cyclical and moderate (same for all examined athletes). Three points-of-time for material sampling against the background of one training process was determined as follows: (1) before training; (2) 20 min after the end of physical exercise; (3) 2–4 h after the end of physical exercise (Figure 1).

**Figure 2:**
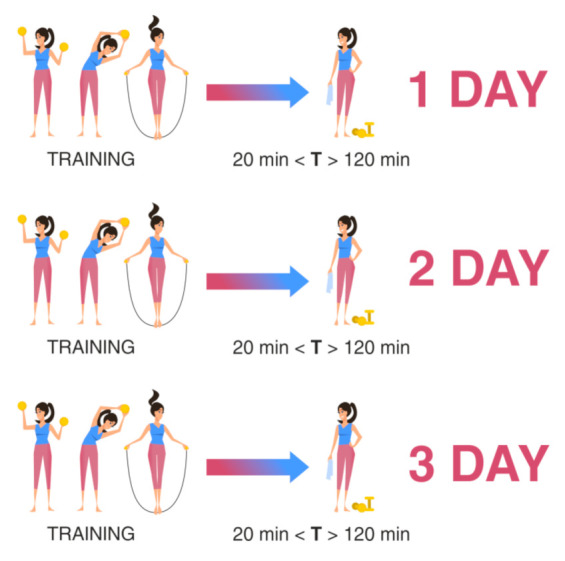
Material sampling for the assessment of the recovery ability of the athlete’s body during the training process. T: time after training.

Scheme II is used to assess the functional state of the athlete against the background of the training process during the entire training cycle with the same nature of loads. Sampling was carried out at three points-of-time for one athlete at the same time interval after the end of the training (at least 20 min and no more than 2 h after the end) on different days of the training process, but at the same daily intervals for all athletes. For example, the sampling of biomaterial was carried out three days in a row, or two days later on the third day, or according to any scheme convenient for the researcher, but it remains the same for all athletes (Figure 2).

Scheme III of material sampling requires a more detailed explanation: biomaterial sampling was carried out at three points-of-time for one athlete at the same time interval after the end of the training (at least 20 min and no more than 2 h after the end) on different days of the training process after the athlete was subjected to physical loads of different powers and durations. For example, aerobic load and anaerobic load, or various loads were used, not in terms of power but in terms of the nature of the exercises. The first day involved swimming with one style, the second day involved swimming with another style, and the third day included a combination of styles. The distances also varied. However, the load pattern and the daily sampling interval remained the same for all athletes.

Research involving athletes must be planned and carried out in accordance with the World Medical Association (WMA) outlined Declaration of Helsinki, ethical principles for medical research involving human subjects, and the Sports National Championships—Medical Minimum Standards (2019–2020). All athletes voluntarily signed informed consent forms to participate in the study. Traditionally, the initial material for obtaining the necessary biological samples (blood, plasma, and serum) is peripheral venous blood. The patient preparation conditions should be followed. Therefore, the patient was in a sitting position while taking the biomaterial sample, and alcohol and food intake immediately before the study was prohibited. The collection of venous blood from athletes at the planned points-of-time during the study should be carried out in the same manner.

Biological materials must be delivered to the laboratory in a timely manner. The optimal storage conditions for biological blood samples include a temperature of +4 °C to +8 °C for 24 h. Freezing of the material is not allowed. Immediately after collection, biological samples were stored in a refrigerator (at a temperature of +4 °C to +6 °C) and then delivered to the laboratory in special transport containers preserving the cold chain. More flexibility in the temperature ranges, time intervals, modes of transportation, and the storage of biomaterials is possible when using the “dry blood spot” technology [23,24].

## 4. Genetic Markers of Sports Success and Occupational Diseases of Athletes

The level of success varies among professional athletes despite the same physical activity during preparation for competitions [25]. In recent decades, numerous scientific studies have been carried out to identify the morphological, anthropometric, physiological, functional, and biomechanical characteristics of elite athletes [26,27]. Until recently, research has relied on associative analysis which involves assessing the correlation between the athletic performance, morpho-physiological variables, and training types of the athletes. In recent years, the research focus has shifted to the genetic analysis and identification of specific polymorphisms in DNA [28,29]. The number of annotated genes potentially associated with athletic performance is increasing annually. Currently, there are approximately 200 known genetic polymorphisms associated with physical activity, of which about 20 variants are associated with the status of an elite athlete [11]. Numerous studies, which have been published over the past decade, have shown that variations in individual genes can influence athletic performance. Some of these studies have shown a link between the allelic frequencies of a particular gene and specific characteristics such as high oxygen consumption (VO_2_max), aerobic capacity, efficiency, or muscle strength [7,30]. An athlete’s ability to perform certain physical activities is determined by the adaptive mechanisms of the circulatory and respiratory systems, skeletal muscles, and other organs. The effectiveness of these mechanisms is genetically determined. It is possible to identify genes that determine a person’s genetic predisposition to a particular sport [28]. Genetic research on athletes has been used to identify specific training methods to increase the load athlete’s able to cope [28]. In addition, optimal results should be achieved with a minimum investment of time and energy, and the prevention of health risks (hypertension, cardiovascular disease, inflammation, and musculoskeletal injury) associated with exercise (Table 1).

In addition, genes may determine the adaptive responses of the athlete’s body to training, injury, and muscle fiber recovery (Table 2).

Thus, numerous studies have shown that the adaptive responses of the athlete’s body can be genetically determined. This includes the level of oxygen consumption, oxidative enzymatic activity, muscle cross-sectional area, and the proportion of slow muscle fibers. Adaptive responses are certainly polygenic, and a single gene may not be responsible for athletic performance. However, it can increase or decrease the athlete’s physical performance capability [82]. Moreover, not all favorable genotypes are present in the same athlete, and differences in athletic performance are the result of a combination of genetic, epigenetic, and psychological factors. Athletic success can be seen as a polygenic characteristic that is controlled by multiple genes where a single polymorphism cannot be responsible for athletic performance. However, it can influence athletes’ performance ability.

Today, a large number of companies offer genetic testing services in order to determine the predisposition to a particular sport, how genetically selected training can improve athletic performance, and how best to prepare for the competition. Some of them are presented in Table 3.

The main genetic markers that are included in the above tests and are associated with athletic performance are presented in Table 4.

Thus, we can conclude that the analysis of differentially expressed genes, as well as genetic polymorphisms, may be of interest for sports medicine as a promising tool to assess athlete’s physiological condition and performance. To date, R&D studies have annotated groups of genes that are probably associated with possible faults during the recovery period of training, i.e., restoration/regeneration of microdamages (*IGF2*), restoration of bone tissue, joints and cartilage (*GDF5*), growth and development of muscle tissue (*TRHR)*, etc. Studied genes can also be associated with the athlete phenotype and determine speed and strength exercises (*ACTN3*), endurance exercise (*PPARA*, *PPARGC1A*, *BDKRB2*), as well as strength exercises (*AGT*, *ACE*, *VDR*, *AMPD1,* etc.). However, these studies have not yet been supported by clinical guidelines and commercially available tests and, hence, are still considered merely as promising genetic markers.

## 5. Proteomic Profile of the Athlete

Proteins are the main components of the cellular metabolic pathways and large-scale studies of the structure, and functions of proteins in the body are useful for identifying the candidate proteins for studying pathological processes and monitoring drug therapy [83]. However, the current number of studies aimed at studying the proteomic composition of athletes is insignificant [22].

When performing physical activities of an aerobic, anaerobic, or mixed nature, with immobilization of a limb under a zero-gravity condition in a state of detraining, the resulting changes in muscle fibers can be characterized by a change in the quantitative and qualitative composition of proteins in biological samples. Such changes are due to transformations at the DNA and RNA levels of the muscle fibers.

The main stress factors that modify the expression of genes of skeletal muscles and nearby structures (satellite cells, vascular endothelium), and affect the plasticity of skeletal muscles, include: (1) mechanical stress, (2) hormonal changes, (3) neuronal activation, and (4) metabolic changes.

The effect of mechanical stress (stretching of muscle fibers) on the neuromuscular apparatus is mediated mainly through integrins and associated signaling pathways. Hormonal changes in skeletal muscles can occur with almost any type of muscle load. Androgens, growth hormones, insulin-like growth factors, spliced forms of insulin, and vitamin D have positive effects on the growth and volume of skeletal muscles (through specific receptors, the expression of a number of genes is triggered). This is mainly due to the activation of muscle satellite cells, while myostatin, interleukin-1 and interleukin-6, glucocorticoids, and tumor necrosis factors are negative regulators of muscle mass and they deactivate satellite cells [84]. The mechanisms that trigger muscle atrophic processes are associated with ubiquitin-mediated protein degradation. An increase in the expression of ubiquitin ligases and atrogin-1 was observed in atrophy-1 [85].

### 5.1. Expression of Proteins in Skeletal Muscles Depending on the Type of Exercise

Training processes aimed at developing endurance or speed-strength qualities are based on external influences of different stimuli that lead to specific structural and metabolic shifts in skeletal muscles [44]. Therefore, during endurance training, there is an increase in the ability of muscles to oxidize lipids and carbohydrates. The content of myoglobin, glycogen, and triglycerides in muscle fibers increases. The size and number of mitochondria also increase. The number of capillaries per muscle fiber and the ability of mitochondria to perform oxidative ATP resynthesis increase. The use of lipids as an energy fuel and selective hypertrophy of slow muscle fibers occurs, along with a slight substitution of fast muscle fibers with slow ones. As a result, the body’s aerobic capabilities increase. On the other hand, training sessions aimed at developing strength, power, or speed had little effect on aerobic performance. Adaptation to sprint and strength training occurs because of a significant increase in the area of the anatomical diameter of skeletal muscles, an increase in the content of creatine phosphate and glycogen as well as glycolytic abilities, an improvement in the buffering properties of muscles, and a decrease in mitochondrial density. This leads to an increase in strength and enhances the ability to perform high-intensity exercises [18]. A single physical activity leads to a change in the expression of hundreds of genes and, accordingly, proteins which come to the initial level after some time (seconds, minutes, hours) [86]. Long-term adaptation to the training of various orientations can be considered as the body’s response to a set of single physical loads. This leads to global changes in the system of gene expression regulation [87].

### 5.2. The Effect of Physical Activity on the Protein Composition of Urine

The practical application of urine proteome analysis is limited by the effects of dilution (daily or one-time sampling) and the presence of several sources of proteins in the urine (glomerular filtration in the kidneys). Nevertheless, the results of the study of the protein composition of urine is used in the clinical diagnosis of kidney diseases, and significant changes in the urine proteome can serve as indicators of physiological changes [88].

Poortmans et al. were the first to analyze various urine proteins, tryptophan-rich prealbumin, albumin, α-1-acid glycoprotein, α-1-antitrypsin, ceruloplasmin, haptoglobin, Gc globulin, transferrin, hemopexin, β-2-glycoprotein I, and γG-globulin, before training and after a marathon [89].

Exercise affects the protein composition of urine. Factors that influence this include temperature, hydration, and the physiological state of the body [90,91]. Gür et al. showed that the amount of excreted protein does not depend on the age and duration of training [92]. For example, muscle damage and hematuria are caused by the same intensity of exercise, leading to the elimination of myoglobin and hemoglobin, respectively. In addition, their fragments are not typical for all athletes. Consequently, in the quantitative analysis of proteins, significant inter- and intra-individual qualitative differences can occur.

The study carried out a comparative analysis of the protein composition of the urine of elite athletes performing various types of exercises [93]. The results of the study showed significant differences both within and between the groups. The work was carried out as part of doping control to detect changes in the protein composition of urine in response to the use of prohibited substances (which included erythropoietin, insulin, or chorionic gonadotropin) and to determine the nutritional status and training status of the athlete.

Generally, proteinuria was found in the samples of every fifth athlete that participated in strength sports, every second athlete that participated in team sports, and almost every endurance athlete. It was not found in samples of the control group. In comparison, strength sports samples showed higher amounts of low molecular weight proteins (transthyretin, CD 59 antigen, GM 2 ganglioside activator, and apolipoprotein A), as well as high molecular weight protein fragments (albumin, transferrin, hemopexin, or IgG fragments) [93].

Another study involved marathon runners. Competitive athletes at rest with an average endurance sports score of 13–20 h per week (triathlon, cycling, and running) were used in control groups. An additional control group of periodically exercising (5 h per week) healthy volunteers was also recruited. No differences were found between the two control groups. Nine out of ten marathon runners had a protein/creatinine ratio of >15 mg/mmol (15–73 mg/mmol). A relative decrease in acidic proteins was observed after exercise which may be related to the changes in erythropoietin levels during doping control. An increase in the level of α-1-acid glycoprotein (orosomucoid) was also observed probably due to increased glomerular filtration and increased levels of transferrin. This may be further associated with changes in glomerular filtration rates or the increased synthesis of transferrin since athletes often show a decrease in iron levels [88].

A number of studies have analyzed the effects of different types of exercise on proteinuria or specific proteins in urine. Proteinuria was found in 61% of male and 66% of female elite badminton players after competition. In addition, the presence of leukocytes (men = 43.5% and women = 50.0%) and erythrocytes (men = 50.0% and women = 21.7%) in urine was investigated [90]. Two-hour karate training (elite athletes, women) sessions did not lead to an increase in the protein/creatinine ratio [94]. Moreover, the effect of different swimming distances (100, 600, and 2000 m) on proteinuria was evaluated. This demonstrated that endurance distances only led to increased albumin levels while shorter distances caused a gradual increase in the tubular marker β2-microglobulin with an increase in swimming speed. In addition, Poortmans et al. found that running 100 km leads to glomerular proteinuria but not tubular proteinuria [95] while intermittent exercise has been reported to have a stronger effect on protein excretion than continuous cycling on an ergometer [96]. Running exercises with 70% of the calculated maximum heart rate under conditions of normoxia and hypoxia, at simulating heights of 2750, 3250, and 3750 m, showed that the amount of excreted protein did not differ significantly under these conditions. In contrast, the specific proteins, albumin and β2-microglobulin, were significantly increased during training under hypoxic conditions [91].

Other factors, such as intake of a special diet or training status, can lead to changes in metabolism of the athletes. This further changes the structure of proteins in urine and requires further elucidation in future studies. Table 5 summarizes the literature data on changes in the proteomic composition of the body of athletes under various training conditions.

The proteome is characterized by high lability and reflects the phenotypic state of the organism at the current time. Proteome studies provide researchers with a wealth of information that allows the identification of candidate protein markers involved in variety of cross-talking biological processes. The role of proteome effect on athlete’s performance should be duly considered because the proteome is more dynamic and agile than genome. It should be noted that ample external factors within a day can substantially change proteins composition and ratio, thus shifting the metabolic prevalence to fatty acids oxidation and carbohydrates uptake. Oddly, the type and duration of training does not always determine excretion, synthesis or degradation rates of proteins. As has been touched on above, the type of exercise in combination with diet typically produces a delayed effect on the proteome and is mostly expressed in the gain of muscle size, fiber stretch capacity and endurance progression. Such effects are caused, in a sense, by the consistent gradual metabolic reprogramming under the auspice of proteome rearrangement. Thus, it can be assumed that the proteome assay mostly reflects the postponed cost of training activity and exercise under the impact of a variety of genetic and epigenetic conditions, including diet, ethnicity, anthropometry and environment. That makes proteome a highly valuable feature in terms of training and activity corrections.

## 6. Metabolome

Modern metabolomics make it possible to identify new predictors and biomarkers for the needs of sports medicine, including the creation of individual recommendations for the training processes for athletes [9]. Metabolomics is a holistic methodological approach for studying the mobility of the composition of low-molecular-weight components in a biological sample in various physiological states of the body by collecting and analyzing big data. By analyzing the content of low-molecular-weight components in tissue samples, cell extracts, and biological fluids (saliva, urine, blood, and sweat), researchers formulated hypotheses about the effects of exercise or nutritional characteristics on the changes in metabolic processes in an athlete’s body [100,101].

Metabolomic studies include targeted and non-targeted analyses. Targeted assays are used to detect metabolites associated with specific biological processes that affect the biological functions of interest. These methodologies have been developed to identify and quantify amino acids, glycerophospholipids, and acylcarnitines associated with cellular energy metabolism. Non-targeted metabolomic or open metabolomics assays use broad analytical scanning techniques in which all detectable metabolites in a biosample can be quantified. Subsequent comparative analysis with the control group made it possible to identify metabolites that are causally and/or significantly associated. One of the biggest challenges of off-target metabolomics is the reliable identification and quantification of the reported metabolites [9].

Currently, the number of metabolomic studies in sports is limited. However, studies indicate that prolonged physical activity and exercise leads to changes in the content of a large number of metabolites in body fluids. Metabolic changes cause lipid mobilization and oxidation. Intense training leads to an increase in the level of endogenous low-molecular-weight components associated with the metabolism of carnitine and long-chain fatty acids and essential fatty acids. Short-term intense exercise causes changes in the levels of metabolites associated with energy production in the muscles [102,103,104].

Modern analytical platforms in the field of metabolomic studies include high-performance liquid chromatography-mass spectrometry (HPLC-MS), gas chromatography-mass spectrometry (GC-MS), and proton nuclear magnetic resonance spectroscopy (1H-NMR). Yan’s research involving the GC-MS analysis of the metabolome of serum samples from 27 athletes revealed changes in the levels of alanine, lactate, beta-dimethylglucopyranoside, pyroglutamic acid, cysteine, glutamic acid, citric acid, free fatty acids, valine, and glutamine [105]. It is likely that energy expenditure increases with increasing physical activity and activates lipolysis and the tricarboxylic acid cycle. Consequently, serum-free fatty acid levels rise during exercise and subsequent recovery [106,107].

The Lehmann group, through an HPLC-MS study, revealed changes in the plasma levels of octanoyl-, decanoyl-, and dodecanoylcarnitines in 29 athletes [108]. In 2017, Karl et al. published an HPLC-MS study in which 25 soldiers took part after a 4-day ski marathon and revealed that blood plasma samples showed an increase in the levels of free fatty acids and metabolites of the tricarboxylic acid cycle; in contrast, the level of monoacylglycerols was decreased [109] (Table 6).

Purine metabolism performs an adaptive function that allows more economical distribution of energy reserves for ATP synthesis during and after training [119]. Studies on urinary metabolite changes in highly qualified long- and medium-distance runners [118,120], sprinters, triathletes [121], and runners with different skill levels [122] found that the lowest hypoxanthine concentration was observed at the peak of the competition while the highest concentration was observed during the transition period.

In a study [123], scientists assessed the effects of continuous exercise on the indices of purine metabolism at rest and after strenuous physical activity over a wide age range. Comparing two opposite training patterns, speed–strength and endurance of athletes (20–90 years old), it was revealed that trained athletes showed lower levels of purine metabolites in plasma than their untrained peers.

In addition, Schranner et al. conducted a systematic review in accordance with the Preferred Reporting Items for Systematic Reviews and Meta-Analyses (PRISMA) guidelines for metabolite levels in blood, urine, or sweat before and within 24 h after strength and endurance exercise. This study identified 196 metabolites that changed significantly within 24 h of exercise. Lactate, pyruvate, tricarboxylic acid cycle intermediates, fatty acids, acylcarnitines, and ketone bodies increased after exercise, while bile acids decreased. At the same time, the concentration of proteinogenic and non-proteinogenic amino acids changes in different directions [124].

Genome-wide association studies for metabolic traits (mGWAS) have revealed hundreds of metabolomic quantitative trait loci (mQTLs) in the general population [125,126]. Identifying new mQTLs in athletes who are exposed to unique environmental conditions, including special diets and intense exercise, is necessary for the discovery of novel biomarkers related to exercise and performance. This unique approach can aid in making more informed selections of athlete candidates and can also provide critical information needed to optimize the balance between the training and recovery periods for each athlete [127]. Al-Khelaifi F. et al. performed GWAS using high-resolution metabolomic profiling of 490 elite athletes and identified common mQTLs, which they later compared with previously identified mQTLs in non-elite athletes. Among the identified mQTLs, endurance-associated metabolites were also present. Two new genetic loci, FOLH1 and VNN1, were reported to be associated with N-acetylaspartylglutamate and linoleoylethanolamide, respectively. In the study of endurance metabolites, one new mQTL that binds androstanediol monosulfate (3-alpha, 17-alpha) and SULT2A1 was identified. The results of this study may play an important role in the selection of athletes who have great potential to become elite athletes in endurance sports. In addition, identifying new mQTLs can also help to identify potential therapeutic targets as they provide direct functional associations between genes and their products that show therapeutic values [128].

In addition to existing methods of studying and monitoring the condition and physical fitness of athletes, metabolic research can also be useful for athletes, coaches, and technical and medical personnel to assess the athletic performance during sports and training. In addition, the results of such studies can be used for the preventive diagnosis of diseases, identification of new talents and predisposition of athletes to injuries. They can also be used to determine the choice of sports, taking into account the individual characteristics of the metabolic profiles of the individuals.

The deeper we delve, the more exciting information we uncover; from the fixed genome given us at birth to the flexible metabolome affected by proteome and rendered by external conditions. Unlike the other above-layered systems, genome and proteome, the metabolome is the most delicate element orchestrating the physical abilities of athletes. It absorbs all up-streamed stimuli and can return immediate effect. Sometimes, it is hard to distinguish changes in metabolome caused by training activity from those caused by abuse of specially designed pharma, since the complex network of metabolic interaction is highly vulnerable to actions. Current knowledge permits us to find the proper balance between training and recovery and to cast the physical activity in terms of regular switching among lipids oxidation, protein synthesis, and carbohydrates uptake. Although this balance is very fragile, it can be sustained for sufficient time to earn the necessary energy generation and proportional improvement of physical abilities. Nonetheless, coaches and athletes should keep in mind that the cost of playing with metabolome is fraught with a dire consequence related to the endocrine system, which succumbs to a much harder salvage.

## 7. The Role of Epigenetics in Athletic Performance

In the process of adaptation of a person to physical activity, the activity of genes changes; some genes are activated, others are inactivated. The observed changes in gene activity underlie cell differentiation in general and in muscle plasticity in particular. Reversible changes in gene activity in the training process of an individual, not associated with a violation of the DNA nucleotide sequence, but leading to the preservation of an inactive or active state of genes in a number of cell generations, are called epigenetic. The inactive state of the gene may be due to a special compact structure of chromatin (heterochromatin), which is formed as a result of the interaction of DNA with specific chromosomal proteins (modification of histones). In some cases, the formation of such a chromatin structure is explained by DNA methylation, and, on the contrary, DNA demethylation may be accompanied by gene activation. Thanks to the latest advances in molecular technology, it has now become possible to determine the epigenetic status of an athlete, which can be passed on from generation to generation. This status makes it possible to identify both active and inactive (methylated) genes of an individual responsible for the development of the functions of muscular, cardiovascular and other body systems, which is important for predicting the body’s athletic capabilities [129].

In addition, specific training protocols are known to affect different signaling pathways and thus affect various characteristics associated with exercise, including angiogenesis, inflammation, muscle recovery, mitochondrial biogenesis, metabolic adaptation, and many others [130,131]. However, the molecular mechanisms of these physiological changes have not yet been determined, and are also subject to significant interindividual variability. However, the variability in adaptation induced by physical activity may be explained in part by epigenetic factors such as noncoding RNAs [132].

MicroRNA (miRNA) levels often change in specific ways in response to various physiological or pathological conditions such as inflammation, cancer, cardiovascular disease, muscle hypertrophy, or exercise [133,134]. Moreover, miRNAs enter the bloodstream and other body fluids in a very stable cell-free form, making them excellent potential diagnostic or prognostic biomarkers related to the rate of change in specific conditions [135]. The authors of [132] characterized the expression patterns of candidate miRNAs reflecting adaptation to exercise at the molecular level to predict physical performance, prevent muscle injury, and monitor recovery. The results of this study show that 8-week explosive strength, hypertrophic strength training, and high-intensity interval training regimens are associated with significant changes in miR-16, miR-21, miR-222, and miR-93 compared to the outcome level in athletic young men [132].

Additionally, there are a large number of studies that have analyzed circulating miRNAs in response to acute or prolonged physical exercise [136,137]. However, further research is urgently needed to determine the potential use of circulating miRNAs as exercise-associated biomarkers, including studying the biological function of circulating miRNAs as physiological mediators of exercise-induced cardiovascular adaptation.

## 8. Discussion

The main goal of the training process in sports, especially at the elite level, is to achieve the greatest cumulative adaptive effect, which should be reflected in an increase in performance indicators, endurance of athletes, and the improvement of sports results. The correctness of the choice of the critical values of the duration, and intensity of the performed exercise/set of exercises was confirmed by the dynamics of molecular and biomedical indicators. In modern sports, there is a continuous increase in the number and the level of competitions, which requires the improvement of existing methods and the creation of new scientific and methodological recommendations to bring the athletes to peak performance during competition season while also prolonging the duration of the periods of peak performance. Taking the scientific achievements and available sports infrastructure into account, it is necessary to improve the recommendations made to young athletes for the early detection of a predisposition to engage in specific sports by using physiological, medico-biochemical, and molecular genetic methods. Scientific research is underway to identify endogenous molecular factors and processes that can be used as predictors of athletic performance, based on the capabilities of the body.

Integration of the results of genetic and metabolomic–proteomic analyses with clinical and biochemical parameters (metadata) will aid in the development of personalized recommendations for effective planning of the training processes. This integration is handled by a new discipline called “-omics” which caters to all needs of sports. Sports science studies the real problems and conditions faced by athletes during sports training and competition. This holistic approach (ex post facto design) involves off-target analysis using a top-down exploration model that requires the analysis of large datasets [9].

In recent years, post-genomic methods of analysis have been increasingly used to search for new markers for analyzing changes in the functional state of the human body. One of the approaches to control the functional state of an athlete undergoing intense physical activity is to assess the complexity of proteins, genes, and metabolites responsible for the implementation of a particular physiological function in the bloodstream during and after physical activity (recovery period). In addition, the approach also aims to compare the results with the profile of a person who does not play sports. The identification of differences in the magnitude of protein expression in longitudinal studies allows the analysis of individual cellular functions and signaling pathways involved in adaptation processes to physical exertion. In some cases, information of this kind helps in the detection of potential biological markers of various pathologies and also helps in describing the processes occurring in the athlete’s body at the molecular level. Proteomic analysis aids in solving particular problems, such as identifying the body’s response to physical activity, assessing the level of fitness, the adequacy of the use of pharmacological and other restorative agents, the role of energy metabolic systems in muscle activity, and the impact of climatic factors. The development of guidelines for the use of proteomic technologies to identify the peculiarities of adaptation of athletes’ bodies to physical activity will significantly increase the effectiveness of the training processes of athletes. One of the most promising areas of research is sports genetics. The genetic code itself, in all likelihood, does not determine the differences in the physical capabilities of a person, nor athletic ability, strength, and health. DNA modifications, primarily methylation and acetylation, ultimately lead to the activation or silencing of genes for a long period of time. It has been proven that physical activity changes the level of gene methylation which helps to suppress tumor growth and affects the development of chronic inflammatory processes. Information about the status of the genetic activity of an elite athlete will help us to understand the general mechanisms of the adaptation of the body to intense physical activity and it also has a pronounced practical application. The results obtained in scientific research can be subsequently used for sports selection, correction of the parameters of the training process, and the diagnosis and treatment of patients who are not professional athletes. Thus, it is advisable to develop a personalized approach based on the analysis of the genome activity of a particular athlete (methylome studies) as part of the basic model of scientific support for high-performance sports. The versatility of metabolomic analysis makes it possible to use it in scientific research in the field of elite sports to assess the specific and integral indicators of biochemical, proteomic-metabolomic, and genetic processes. The control of the functional state of an athlete undergoing intense physical activity involves the assessment of the metabolomic profile, during and after physical activity (recovery period), and comparing it with the profile of a non-athlete.

## 9. Conclusions

An objective assessment of the predisposition of an individual to a particular sport, which is necessary for the effective selection and orientation of children and adolescents for sports, should be based on comprehensive research, including the definition and analysis of morphological, functional, biomechanical, pedagogical, psychological and, in the near future, molecular genetic criteria.

It is imperative to continue scientific research into the exercise-mediated changes observed in the multi-omics (proteins, genes, metabolites) composition under different types of exercise conditions. The observed variations will help systematize the molecular changes for specific groups and are important to expand the knowledge about exercise-induced changes, including drug testing, sports physiology, and the detection of biomarkers in diseases, as levels of certain proteins can be altered after exercise. The use of modern molecular genetic methods in scientific research and sports practice will lead to a deepening of our fundamental knowledge. It will also be of great practical importance as it can aid in enhancing the sports’ results, allow for the formulation of practical recommendations on the organization of personalized training, reduce the financial costs of training athletes, and decrease the risk of athletes and non-athletes developing chronic diseases and pathological conditions.

## Figures and Tables

**Table 1:** Genes associated with athletic performance.

Gene	Function	Biological Process (Gene Ontology)	Exercise Type	Ref.
*AGT*	regulation of blood pressure, heart function, vascular smooth muscle growth, sympathetic, neuromuscular systems	regulation of blood pressure (GO:0008217)	strength exercises	[30,31,32,33]
*ACE*	regulation of blood pressure and electrolyte balance	regulation of blood pressure (GO:0008217)	strength exercises	[34,35,36]
*ACTN3*	contraction of skeletal muscle during exercise through structural and metabolic changes	muscle system process (GO:0003012)	speed and strength exercises	[37,38]
*PPARA*	fatty acid metabolism	positive regulation of fatty acid oxidation (GO:0046321)	endurance exercise	[39,40]
*PPARGC1A*	mitochondrial biogenesis	regulation of developmental process (GO:0050793)	endurance exercise	[41,42,43]
*VEGFA*	skeletal development, regulation of vascular endothelial growth factor	regulation of developmental process (GO:0050793)	aerobic exercise	[44,45]
*VDR*	calcium homeostasis	homeostatic process (GO:0042592)	strength exercises	[46,47]
*BDKRB2*	vascular permeability, hypotension, smooth muscle contraction and glucose homeostasis	muscle system process (GO:0003012)	endurance exercise	[48,49]
*NFE2L2*	mitochondrial biogenesis	positive regulation of reactive oxygen species metabolic process (GO:2000379)	aerobic exercise	[50,51]
*MSTN*	myoblast cell proliferation	response to testosterone (GO:0033574)	strength exercises	[52]
*FTO*	energy exchange	regulation of developmental process (GO:0050793)	sports in heavy weight	[53,54]
*AMPD1*	regulation of the formation of ATP from ADP	response to organic substance (GO:0010033)	strength exercises	[55,56]
*NOS3*	restoration and regeneration of the myocardium, glucose metabolism, ATP synthesis and oxygen consumption by skeletal muscles	regulation of developmental process (GO:0050793)	aerobic exercise, strength exercises	[57,58]
*SLC16A1*	facilitated diffusion of lactate	monocarboxylic acid transport (GO:0015718)	strength exercises	[59,60]

**Table 2:** Genes associated with athlete’s health risks.

Gene	Function	Phenotype	Ref.
*TNC*	regulation of inflammatory processes	weakness of tendons, trauma and tendinopathies	[61]
*IGF* *2*	restoration/regeneration of microdamages	muscle damage in men	[62,63]
*LRP5*	osteoblast differentiation	low bone mineral density, risk of osteoporosis	[64,65]
*MMP3*	extracellular matrix homeostasis, through modulating the structural and biological integrity of the tendons and ligaments	non-contact anterior cruciate ligament rupture risk, Achilles tendinopathy risk	[66,67]
*TRHR*	stimulates exocytosis of thyroid-stimulating hormone into the blood	growth and development of muscle tissue	[68,69]
*IL6*	regulation of intercellular interactions secreted during inflammation	adaptive training responses, muscle fiber recovery	[70,71]
*ADRB2*	receptors for catecholamines (adrenaline, norepinephrine), regulation of energy expenditure	individual response to diet and physical activity	[72,73]
*COL1A1*	the main organic component of the bone matrix	injury protection and recovery	[74,75]
*COL5A1*	structural component of tendons and other connective tissues, etiopathogenesis of tendinopathy	etiopathogenesis of tendinopathy	[76,77]
*CRP*	regulation of exercise-induced inflammatory disorders	increased physical activity	[78,79]
*GDF5*	participation in the formation of joints	restoration of bone tissue, joints and cartilage, the risk of osteoarthritis of the joints	[80,81]

**Table 3:** Some companies that offer genetic testing services to determine the predisposition to a particular sport.

Resource	Test	Reference	Accession Date
Genorama	Athletic Performance test	https://www.genorama.com	11 June 2021
CrossDNA	Sport DNA testing	https://crossdna.com/en	4 June 2021
Sports Gene Llc	Genetic Test of Athletic Abilities	https://www.sportsgene.ee/en	13 May 2021
24Genetics	DNA sport test	https://24genetics.com/en	11 May 2021

**Table 4:** Genetic markers of sports success and professional pathologies of athletes *.

Gene	Genotype	Gene	Genotype	Gene	Genotype
*Sport Profile*		*Muscle Profile*		*PPARGC1A*	CC
Resistance		Muscular strength		*ADRB2*	GG
*PPARGC1A*	CC	*PPARD*	TT	*PPARD*	TT
*ACE*	GG	*PPARGC1A*	CC	Injury Risk	
*NFIA*	GG	*HIF1A*	CC	General risk of injury	
*HIF1A*	CC	*GDF8*	TT	*GDF5 AG*	AG
Power		*IGF1*	TT	*COL1A1*	CC
*ACE*	GG	*SLC30A8*	TC	*IL6*	CC
*IGF2BP2*	TG	*CCL2*	AG	*CRP*	CC
*NOS3*	GG	Muscle response to resistance training		Risk of injury to joints	
*PPARG*	CC	*BMP2*	AA	*GNL3*	AG
*AGT*	AG	*IL15RA*	TC	*FTO*	TT
*PPARA*	CG	*INSIG2*	CG	*SUPT3H*	GG
*VEGFA*	CG	Skeletal Muscle Performance		*IL1A*	AG
*VDR*	CG	*UCP2*	TC	Risk of overload fracture	
*PPARGC1A*	CC	Muscular fatigue	*RIN3*	СС	
*HIF1A*	CC	*HNF4A*	AG	*C17ORF53*	AA
Strength		*NAT2*	AA	*MEPE*	GG
*INSIG2*	CG	Muscle regeneration capacity		*ZBTB40*	GG
Cardio capacity		*IL1B*	GG	Risk of ruptured tendons and ligaments	
*NPY*	TT	*IL1B*	AA	*COL1A1*	CC
*NOS3*	CC	*Metabolic Profile*		*MMP3*	CC
*ADRB1*	GC	Global benefit of the sport in your body		*GDF5*	AG
*APOE*	TT	*CETP*	CC	*COL12A1*	TT
*APOE*	CC	*BDNF*	TC	Cardiovascular profile	
Aerobic capacity		Benefit of Exercise in Insulin Sensitivity		*EDN1*	TG
*NFIA-AS2*	*GG*	*LIPC*	TT	*GG*	TC
*RGS18*	*AG*	Benefits of Exercise in Cholesterol		*GNAS*	TC
*ACSL1*	*AA*	*FTO*	NOS3	*ADD1*	GG
Resilience		*FTO*	CC	-	-
*IL6*	CC	Metabolic efficiency		-	-
*CRP*	CC	*AMPD1*	GG	-	-
*SOD2*	AG	*PPARA*	CG	-	-

* Adapted from 24 Genetics’ DNA sport test.

**Table 5:** Proteomic studies of athletes.

Method	*N* Proteins	Biomaterial	Exercise Type	Ref.
nanoLC-MS	60	dry blood spot	running and cycling	[21]
off-gel LC-MS/MS	92	biopsy	bicycle riding	[97]
MALDI-TOFMS	52	plasma	spartathlon race	[21]
nano-UPLC-Orbitrap MS	5	urine	marathon	[88]
(Q)-PCR	>250	biopsy	cycling and powerlifting	[98,99]
2D-PAGE	12	urine	endurance, team sports, strength sports	[93]

**Table 6:** Changes in the content of endogenous metabolites in biosamples of athletes.

Metabolite	Sport	Fold Changes	Biosample	Pathway (KEGG)		Ref.
2-hydroxybutyrate, 2-oxoisocaproate	sprint sessions	▲	urine	C05984 Propanoate metabolism		[110]
	leg press and knee extension exercises		serum	C00233 Propanoate metabolism		[111]
3-methyl-2-oxovalerate	sprint sessions	▲ recovered 1 h after training	urine	C06008 Metabolic of Lipids		[110]
3-hydroxyisobutyrate				C06001 Valine, leucine and isoleucine degradation		[110]
2-oxoisovalerate				C00141 Valine, leucine and isoleucine biosynthesis		[110]
3-hydroxybutyrate				C11551 Metabolic pathways		[110]
2-hydroxyisobutyrate				C21297 Pantothenate and CoA biosynthesis		[110]
alanine	sprint sessions	▲ recovered 1 h after training	urine	C00041 Alanine, aspartate and glutamate metabolism		[110]
	boating	▲	serum			[105]
	leg press and knee extension exercises	▲	serum			[111]
pyruvate	sprint sessions	▲ recovered 1 h after training	urine	C00022 Glycolysis/Gluconeogenesis		[110]
	leg press and knee extension exercises	▲	serum			[111]
fumarate	sprint sessions	▲ recovered 1 h after training	urine	C00122 TCA cycle		[110]
lactate	sprint sessions	▲ recovered 1.5 h after training	urine	C00186 Pyruvate metabolism		[110]
	boating	▲	serum			[105]
acetate	sprint sessions	▲ recovered 1.5 h after training	urine	C00033 Taurine and hypotaurine metabolism		[110]
urate	sprint sessions	▼,▲ in 1.5 h and recovered 2 h after training	urine	C00366 Purine metabolism		[110]
valine	sprint sessions	▼,▲ in 1.5 h after training	urine	C00183 Valine, leucine and isoleucine degradation		[110]
	leg press and knee extension exercises	▼	serum			[111]
isoleucine	sprint sessions	▼,▲ in 1.5 h after training	urine	C00407 Valine, leucine and isoleucine degradation		[110]
succinate	sprint sessions	▼,▲ in 1.5 h after training	urine	C00042 TCA cycle		[110]
	leg press and knee extension exercises	▲	serum			[111]
citrate	sprint sessions	▼, ▲ in 1.5 h after training	urine	C00158 TCA cycle		[110]
trimethylamine				C00565 Methane metabolism		
N-oxide				C01104 Methane metabolism		
tyrosine				C00082 Tyrosine metabolism		
formate				C00058 Pyruvate metabolism		
β-aminoisobutyric acid	short-term intense exercise	▲	plasma	C01205 Pyrimidine metabolism		[112,113]
kynurenic acid	bicycle race	▲		C01717 Tryptophan metabolism		[112,114]
12,13-Dihydroxy-9Z-Octadecenoic Acid	Acute exercise (cycle ergometer)	▲	serum	C01595 Linoleic acid metabolism		[112,115]
palmitoylcarnitine	marathon competition	▲ recovered 72 h after training	plasma	C02990 Fatty acid metabolism		[116]
free carnitine				C00318 Bile secretion		[116]
acetylcarnitine	marathon competition	▲ recovered 72 h after training	plasma	C02571 Insulin resistance		[116]
	ski march	▲				[109]
arginine	marathon competition	▼ sharp ▲ within 72 h. of recovery	plasma	C00062 Arginine and proline metabolism		[116]
glutamate				C00025 Alanine, aspartate and glutamate metabolism		
ornithine	marathon competition	▼ ▼ sharp ▲ within 72 h of recovery	plasma	C00077 Arginine and proline metabolism		[116]
	leg press and knee extension exercises	▼	serum			[111]
phosphatidylcholine	marathon competition	▼ sharp ▲ within 72 h of recovery	plasma	C00157 Glycerophospholipid metabolism		[116]
lysophosphatidylcholine				C04230 Glycerophospholipid metabolism		
succinic acid	football	▼	urine	C00042 TCA cycle		[117]
glutaric acid				C00489	Fatty acid degradation	
aminomalonic acid				C00872 Taurine and hypotaurine metabolism		
3-hydroxy-3-methyl				C03761 Citrate cycle		
ribose				C08353 Ribose biosynthesis		
guanidinosuccinic acid				C03139 Arginine biosynthesis		
quinolinic acid				C03722 Tryptophan metabolism		
xanthurenic acid				C02470 Tryptophan metabolism		
palmitate (16:0)	ski march	▲	plasma	C00249 Fatty acid biosynthesis		[109,111]
3-hydroxybutyrate				C01089 Synthesis and degradation of ketone bodies		
diacylglycerol oleoyl-oleoyl-glycerol palmitoleoyl-arachidonoyl-glycerol		▼		C00165 MAPK signaling pathway		
2-hydroxybutyrate				C05984 Propanoate metabolism		
2-oxoarginine				map00330 Amino acid metabolism		
cysteine	boating	▲	serum	map00270 Amino acid metabolism		[105]
glutamic acid		▼		C00025 Alanine, aspartate and glutamate metabolism		
pyroglutamic acid						
hippurate	run	▲	plasma	C01586 Phenylalanine metabolism		[108]
carnitine and its derivatives				C00487 Lysine degradation		
hypoxanthine				C00262 Purine metabolism		
isoleucine		▼		C16434 Valine, leucine and isoleucine degradation		
leucine				C00123 Valine, leucine and isoleucine degradation		
lysine				C00047 Lysine biosynthesis		
hypoxanthine	run	▼	plasma	C00262 Purine metabolism		[118]

▲—increase; ▼—decrease.

## Data Availability

This is a review paper that collected from public data listed in the “Reference” and from open accessible web-sources listed in the Table 3 (all web-link were valid upon this Review paper has been published).
